# The prognostic value of COX-2 in predicting metastasis of patients with colorectal cancer: A systematic review and meta analysis

**DOI:** 10.1016/j.heliyon.2023.e21051

**Published:** 2023-10-14

**Authors:** Andriana Purnama, Kiki Lukman, Reno Rudiman, Dwi Prasetyo, Yoni Fuadah, Prapanca Nugraha, Valeska Siulinda Candrawinata

**Affiliations:** a Division of Digestive Surgery, Department of Surgery, Padjadjaran University, Bandung, Indonesia; b Division of Pediatric Gastroenterology, Department of Pediatric, Faculty of Medicine, Padjadjaran University, Bandung, Indonesia; c Department of Forensic and Medicolegal, Faculty of Medicine, Padjadjaran University, Bandung, Indonesia; d Faculty of Medicine, Pelita Harapan University, Tangerang, Indonesia

**Keywords:** COX-2, Metastasis, Colorectal cancer, Prognosis

## Abstract

**Introduction:**

COX-2 is overexpressed in colorectal tumour tissue relative to the healthy colonic mucosa, thus we investigated the prognostic significance of COX-2 in determining the metastasis of patients with colorectal cancer.

**Methods:**

PubMed, EMBASE, and Cochrane Library were searched using the following terms colorectal cancer, colon cancer, rectal cancer, colorectal carcinoma, Cyclooxygenase-2, and prognosis to identify articles providing information on the prognostic importance of COX-2 in adult patients with metastatic colorectal cancer. Review papers, non-research letters, comments, case reports, animal studies, original research with sample sizes of fewer than 20, case reports and series, non-English language articles, and pediatric studies (those under the age of 17) were excluded. The Newcastle Ottawa Scale (NOS) was used to assess the credibility of the included studies. The full texts were evaluated and this study complied with the terms of the local protocol and the Helsinki Declaration.

**Results:**

Eight relevant studies were included in this review involving 937 patients. The meta-analysis revealed that COX-2 expression is associated with lymph node invasion (RR 1.85 [1.21, 2.83], P = 0.005, I2 = 88 %) and liver metastasis (RR 4.90 [1.12, 21.57], P = 0.04, I2 = 42 %), but not with venous dissemination (RR 1.48 [0.72, 3.03], P = 0.28, I2 = 87 %).

**Conclusion:**

COX-2 expression is associated with lymph node invasion in colorectal cancer but further studies are required to determine the prognostic significance of COX-2 expression in determining metastasis status for colorectal cancer patients.

## 1 Introduction

Colorectal cancer (CRC) accounted for 1.9 million new cases and 0.9 million fatalities in 2020 [1]. It is the second most frequent disease in women and the third most common cancer in men. The incidence of CRC has increased recently and accounts for approximately 10 % of all cancers and is the second most common cause of cancer mortality. Hence, CRC is a major global public health concern in terms of morbidity, death, and medical services, including rising expenses [1].

The main reason for death from colorectal cancer is metastasis and the invasion ability of tumour cells predicts the development of new metastases. An essential stage in tumour invasion is proteolysis of the basement membrane, which comprises laminin, fibronectin, type IV collagen, and proteoglycan. Indeed, MMP activity is significantly elevated in Caco-2 colon cancer cells that overexpress Cyclooxygenase-2 (COX-2) [2,3]. The story begins with the discovery of COX enzymes, which are responsible for the synthesis of prostaglandins. In 1971, it was established that there were two distinct forms of COX enzymes: COX-1 and COX-2. COX-1 is constitutively expressed in many tissues and plays a role in maintaining normal physiological processes, while COX-2 is induced in response to inflammatory stimuli [4]. Sulindac sulphide therapy can reverse increased invasiveness and PGE2 production, establishing a clear connection between COX-2 and MMP activation [5]. According to Murata et al. human gastric cancer may more likely develop lymphatic invasion and metastases if COX-2 is overexpressed [6].

The future of CRC research involves a shift towards personalized medicine. Understanding the genetic and molecular profiles of individual CRC tumors can help tailor treatments, including the use of COX-2 inhibitors, to specific patients [4]. The role of COX in tumour growth and development has received much attention. Cyclooxygenase, which has two distinct isoforms (COX-1 and COX-2), functions as a rate-limiting enzyme in the production of prostanoids [7]. COX-2 is increased in response to cytokines, growth factors, and tumour promoters. Inflammation, wound healing, and carcinogenesis have all been linked to their pathophysiologic role [8,9]. Moreover, COX-2 is overexpressed in the tumour tissue of colorectal cancer relative to the healthy colonic mucosa [10].

Numerous retrospective investigations have examined the possibility that COX-2 overexpression may be a predictive factor for survival in individuals with colorectal cancer [[11], [12], [13]], showing that poor prognosis is associated with high COX-2 levels in tumour tissue, whether in the tumour cells or the stroma compartment [12,13]. As COX-2 is an inflammatory marker, it can be found in the blood. Inflammatory peritumoral cells, primarily lymphocytes, and cancer cells can also secrete COX-2 [7]. The administration of steroid and non-steroid anti-inflammatory drugs (NSAIDs) to cancer patients may inhibit the levels of peripheral blood COX-2 released by tumors, resulting in a change in serum levels [14,15]. Thus, establishing if COX-2 expression is a predictive marker in CRC is necessary.

Previous studies on COX-2 as a predictor for CRC outcomes have yielded inconsistent findings due to differences in patient characteristics, research methods, and outcome measures [16,17]. Inadequate consideration of influential factors and publication bias further contribute to the varied results. Conducting a comprehensive meta-analysis can help address these discrepancies by systematically analyzing existing data, accounting for variables, and offering a clearer assessment of COX-2 potential as a predictor in CRC outcomes [16,18]. This meta-analysis of all relevant published studies regarding the association between COX-2 and CRC survival aimed to determine the prognostic significance of COX-2 in determining the metastasis of CRC patients.

By analyzing methodological differences, addressing confounding variables, and reconciling conflicting results, the review anticipates shedding light on the true predictive value of COX-2 in CRC outcomes. These findings could guide future research by highlighting the gaps, refining methodologies, and suggesting new avenues for investigating the potential of COX-2 as a valuable predictor in CRC prognosis and treatment strategies. However, several potential confounding factors associated with COX-2 in predicting CRC outcomes could exert influence on the results of the meta-analysis, including variations in cancer stage, diverse treatment regimens, patient-specific characteristics, and the presence of other prognostic markers.

## 2 Methods

### 2.1 Database and literature search

PubMed, EMBASE, and Cochrane Library were searched using the following terms colorectal cancer, colon cancer, rectal cancer, colorectal carcinoma, Cyclooxygenase-2, prognosis, and outcome from 18 to 20 February 2023 to identify articles related to the prognostic value of COX-2 for CRC patients (Table 1). The initial search and screening were completed by two independent authors and studies providing information on prognostic importance involving adult patients with metastatic CRC were included. Any discrepancies were resolved by discussion with other authors.

**Table 1:** Detailed search terms used from PubMed, Cochrane Library, and EMBASE.

Database	Keywords	Total studies extracted
PubMed	(“prognosis" [MeSH Terms] OR “prognosis" [All Fields] OR “prognoses" [All Fields] OR (“outcome" [All Fields] OR “outcomes" [All Fields])) AND (“colorectal neoplasms" [MeSH Terms] OR (“colorectal" [All Fields] AND “neoplasms" [All Fields]) OR “colorectal neoplasms" [All Fields] OR (“colorectal" [All Fields] AND “cancer" [All Fields]) OR “colorectal cancer" [All Fields] OR (“colorectal neoplasms" [MeSH Terms] OR (“colorectal" [All Fields] AND “neoplasms" [All Fields]) OR “colorectal neoplasms" [All Fields] OR (“colorectal" [All Fields] AND “carcinoma" [All Fields]) OR “colorectal carcinoma" [All Fields])) AND (“metastasi" [All Fields] OR “neoplasm metastasis" [MeSH Terms] OR (“neoplasm" [All Fields] AND “metastasis" [All Fields]) OR “neoplasm metastasis" [All Fields] OR “metastasis" [All Fields] OR (“metastasation" [All Fields] OR “metastasic" [All Fields] OR “metastasing" [All Fields] OR “metastasize" [All Fields] OR “metastasized" [All Fields] OR “metastasizes" [All Fields] OR “metastasizing" [All Fields] OR “metastasization" [All Fields] OR “metastasizes" [All Fields] OR “metastasizing" [All Fields] OR “neoplasm metastasis" [MeSH Terms] OR (“neoplasm" [All Fields] AND “metastasis" [All Fields]) OR “neoplasm metastasis" [All Fields] OR “metastase" [All Fields] OR “metastases" [All Fields] OR “metastasize" [All Fields] OR “metastasized" [All Fields])) AND (“COX-2" [All Fields] OR (“cyclooxygenase 2" [MeSH Terms] OR “cyclooxygenase 2" [All Fields]))	113
Cochrane	prognosis OR outcome AND Colorectal cancer OR colorectal carcinoma AND Metastasis OR metastases AND COX-2 OR cyclooxygenase-2	287
EMBASE	(((‘prognosis'/exp OR prognosis OR ‘outcome'/exp OR outcome) AND (‘colorectal cancer'/exp OR ‘colorectal cancer’ OR (colorectal AND (‘cancer'/exp OR cancer))) OR ‘colorectal carcinoma'/exp OR ‘colorectal carcinoma’ OR (colorectal AND (‘carcinoma'/exp OR carcinoma))) AND (‘metastasis'/exp OR metastasis) OR 'metastases'/exp OR metastases) AND ‘cox 2′ OR ‘cyclooxygenase 2'/exp OR ‘cyclooxygenase 2′ AND [embase]/lim NOT ([embase]/lim AND [medline]/lim) AND ‘article'/it	7920

### 2.2 Study selection

After the initial search, duplicate studies were excluded and all authors separately reviewed the titles and abstracts of relevant articles. The full texts were evaluated against the inclusion and exclusion criteria. Articles were included in this systematic review if they fulfilled the following criteria: 1) Population: adult CRC patients; 2) Intervention: Positive/high COX-2 expression; 3) Comparison: Negative/low COX-2 expression; 4) Primary outcome: lymph node metastasis and venous dissemination; secondary outcome: liver metastasis of the tumour, both of which must be confirmed by immunohistochemical staining; 5) Type of studies: observational. Studies that did not provide the aforementioned were excluded. Review papers, non-research letters, comments, case reports, animal studies, original research with sample sizes of fewer than 20, case reports and series, non-English language articles, and studies on the pediatric population (those under the age of 17) were also excluded. The study complied with the terms of local protocol and the Helsinki Declaration. This systematic review and meta-analysis adhered to the Preferred Reporting Items for Systematic Reviews and Meta-Analyses (PRISMA) [19] and was registered in PROSPERO with the following ID: CRD42023403067.

### 2.3 Data extraction and quality assessment

Each author worked individually to extract the data, which was recorded using a standardised form containing author information, year, research design, age, gender, stage of the malignancy, treatment received before surgery, length of follow-up, testing procedure, cutoff value, and overall survival. The primary study authors were not contacted to ask for extra or unreported data. The Newcastle Ottawa Scale (NOS) was applied to assess the credibility of the included studies, which consists of a total quality rating of nine stars [20]. The authors solved any disagreements that occurred through discussion.

### 2.4 Statistical analysis

Review Manager V.5.4 (Cochrane Collaboration) was used to conduct the meta-analysis. The Mantel-Haenszel formula is a statistical method used for dichotomous variables (i.e., variables that have two possible outcomes, such as success/failure or presence/absence). It calculates risk ratios (RRs) and their corresponding 95 % confidence intervals (CIs). RRs represent the likelihood of an event occurring in one group compared to another. Confidence intervals provide a range of values within which the true population parameter (in this case, the true RR) is likely to fall with a certain level of confidence (usually 95 %). Narrower CIs indicate greater precision. In hypothesis testing, p-values assess the statistical significance of an observed effect. Two-tailed p-values are commonly used and indicate whether an effect is statistically significant in either direction (i.e., it could be higher or lower than expected by chance). A significance level of 0.10 is specified for assessing heterogeneity, which suggests a more relaxed criterion for considering differences between studies as significant. Heterogeneity refers to the degree of variability or diversity among the results of individual studies included in the meta-analysis. In this case, a significance level of 0.10 is used to assess heterogeneity, suggesting that statistical tests for heterogeneity will be conducted with a relatively relaxed threshold. Publication bias occurs when the publication of research results is influenced by the direction or statistical significance of the findings. To assess the risk of publication bias, inverted funnel-plot analysis is used. Funnel plots visually display the relationship between effect size and study precision, and an asymmetric funnel plot can suggest potential bias in the literature.

## 3 Results

**Fig. 1:**
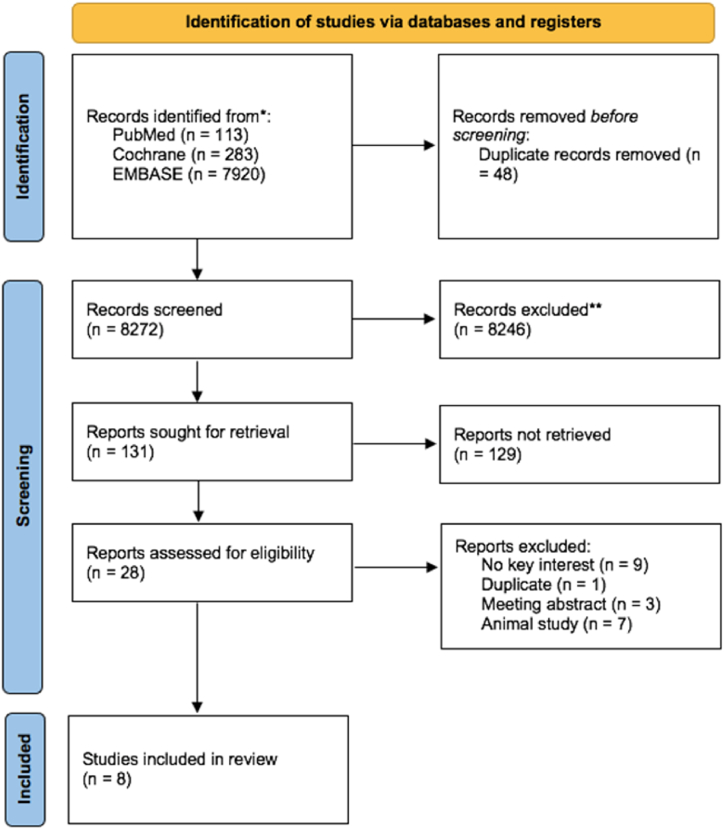
PRISMA flow diagram.

The initial search retrieved 8320 records from three unique electronic databases, and after screening and duplicate removal, twenty-one potential articles remained. After screening the titles and abstracts, 8251 studies were excluded. Following a review of twenty-three full-text articles for eligibility, twenty papers were deemed ineligible due to being conference abstracts (n = 3), duplicate studies (n = 1), studies that failed to disclose the key interests (n = 9), and research involving animals (n = 7) (Fig. 1). Eight retrospective observational studies were included in this meta-analysis involving 937 patients [[21], [22], [23], [24], [25], [26], [27], [28]], their baseline characteristics and quality assessments are shown in Table 2.

**Table 2:** Summary of cohort from included studies (n = 937).

Study ID, study quality by NOS	Total cohort (n)	Male (n)	Age (years)	Location		Lymph node metastasis (n)	Hematogenous metastasis (n)	Distant metastasis (n)	COX-2 positive (n)	COX-2 sampling location
				Colon	Rectal					
Chen 2001, 6	17	13	–	13	4	–	–	–	17	Tissue
Wu 2003, 7	139	70	59 (22–89)	54	85	44	32	–	118	Tissue
Soumaoro 2006, 7	109	67	60 ± 10	64	45	67	97	–	109	Tissue
Konno 2002, 7	56	36				23	18	Liver: 18	14	Tissue
Yamauchi 2002, 7	232	140		144	88	157	28	Liver: 1 Lung: 3 Local: 1 Peritoneum:0 Bone: 0	166	Tissue
Pancione 2009, 6	72	44	70.3 ± 12.9	19	53	21	–	20	39	Tissue
Yamac 2005, 7	83	47		53	30	39	26		52	Tissue
Zhou 2018, 7	229	133		106	123	146		29	97	Tissue

### 3.1 Prognostic significance of COX-2 for CRC patients

**Fig. 2:**
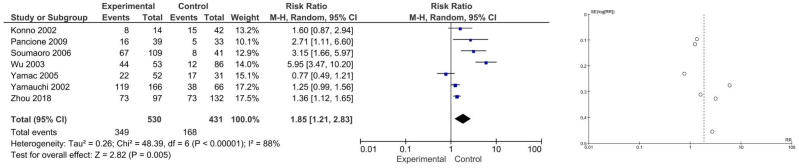
Forest plot and funnel plot for lymph node invasion.

**Fig. 3:**
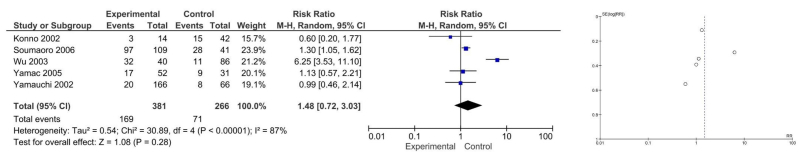
Forest plot and funnel plot for venous dissemination.

**Fig. 4:**
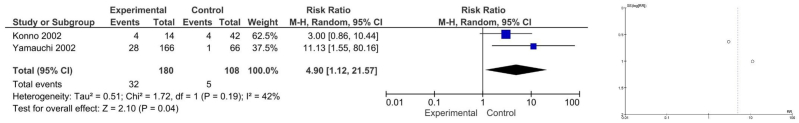
Forest plot and funnel plot for liver metastasis.

COX-2 expression is associated with lymph node invasion (RR 1.85 [1.21, 2.83], P = 0.005, I2 = 88 %) and liver metastasis (RR 4.90 [1.12, 21.57], P = 0.04, I2 = 42 %), but not with venous dissemination (RR 1.48 [0.72, 3.03], P = 0.28, I2 = 87 %). A detailed forest plot of COX-2 expression in determining lymph node, venous dissemination, and liver metastasis for CRC is provided in Fig. 2, Fig. 3, Fig. 4, respectively.

### 3.2 Risk of publication bias

The inverted funnel plot had a qualitatively symmetrical shape indicating a low likelihood of publication bias. Detailed funnel plots of COX-2 expression in determining lymph node, venous dissemination, and liver metastasis for CRC patients are displayed in Fig. 2, Fig. 3, Fig. 4, respectively.

## 4 Discussion

### 4.1 Study rationale

COX-2 is a fast-acting rate-limiting enzyme which transforms arachidonic acid into prostaglandins and thromboxanes, such as PGE2. According to recent studies, PGE2 accelerates the onset and spread of CRC [[29], [30], [31]]. For instance, Src transactivates EGFR to facilitate PGE2-mediated invasion [31]. PGE2 also promotes the invasion of CRC via PI3K. Furthermore, intestinal cell adhesion can be impacted by COX-2 overexpression, which in turn increases matrix metalloproteinase activity and cancer invasion [32]. In both human and mouse models, COX-2 inhibition can prevent the spread of CRC and CRC is associated with loss of cell-cell contact and invasion with c-Met, the hepatocyte growth factor receptor, transactivated by PGE2 via EGFR [32,33].

### 4.2 Key results and interpretation

This meta-analysis found that COX-2 expression is associated with lymph node invasion and liver metastasis in CRC but not with venous dissemination. These findings are consistent with numerous immunohistochemical studies and mRNA analyses of multiple human malignancies, including prostate, gastric, lung, breast, and oesophagal cancers [[33], [34], [35]]. The frequent colocalisation of VEGF-C suggests that a mechanism may be controlling how these two genes are expressed in tumour cells. Su et al. revealed that cells transfected with the Cox-2 gene or exposed to prostaglandin E2 significantly increased VEGF-C mRNA and protein levels [36]. Moreover, endogenous VEGF-C protein levels were significantly reduced after these cells were treated with a COX-2-specific inhibitor. Thus, it is hypothesised that the EP1 prostaglandin receptor and the HER-2/Neu-dependent pathway are involved in the up-regulation of VEGF-C by COX-2 [37] and that the EP2 prostaglandin receptor mediates COX-2 action in angiogenesis.

Angiolymphangiogenesis, the process that results in the development of new blood or lymphatic vessels, has been linked to the survival, proliferation, and dissemination of cancer cells [38]. Cells must leave the primary tumour and enter the lymphatic or vascular network to travel to other organs. Intratumoral lymphangiogenesis has been demonstrated in a nude mouse model of spontaneous breast cancer metastasis using human breast cancer cells transfected to express VEGF-C [38,39]. Also, VEGF-C is a strong promoter of tumour lymphangiogenesis, which increases the metastatic dissemination of breast cancer cells to the lymph nodes and lungs. In addition, COX-2 is involved in tumour neovascularisation, invasiveness, and metastatic potential, including CRC [40]. Strong experimental evidence supports these claims that angiogenesis and lymphangiogenesis inhibitors have anticancer and anti-metastatic effects [[39], [40], [41]].

The release of chemoattractants that stimulate the migration of liver sinusoidal endothelial cells and hepatic stellate cells is facilitated by COX-2 activation in cancer cells [42,43]. Interestingly, PGE2 promotes endothelial cell migration and tube formation [44]. Our meta-analysis showed a statistically significant association between COX-2 and liver metastasis status in CRC patients, which may be mediated by decreased liver sinusoidal endothelial cell migration after stimulation with CLX-treated C26 secretomes [45]. PGE2 and VEGF in the tumour microenvironment may be produced by C26 cells and hepatic stellate cells, are decreased in CLX-treated animals and are associated with a decrease in tumour formation [46]. PGE2 and VEGF levels decrease in CLX-treated mice, which may impede myeloid-derived suppressor cell recruitment and differentiation, as observed in other cancer models following COX-2 inhibition [47,48].

### 4.3 The utilization of COX-2 for CRC patients

COX-2 may be employed for risk stratification to establish the best course of treatment for CRC patients depending on their probability of developing metastases. It is recommended that future immunohistochemical investigations to build prognostic models include COX-2 as an immunohistochemical marker. Also, as several publications mentioned the possibility of COX-2 measurement in serum [12,13], future studies should examine serum COX-2 levels in CRC patients to determine a cut-off value for determining which patients may have worse outcomes than the others. By incorporating COX-2 status into clinical decision-making, oncologists could optimize treatment selection and intensity, enhancing patient outcomes. For instance, individuals with high COX-2 expression levels might be considered for targeted therapies aimed at reducing COX-2 activity and dampening inflammatory pathways that promote metastasis [4,49,50]. Conversely, patients with low COX-2 expression could be spared from unnecessary aggressive treatments, minimizing potential side effects [50] Furthermore, the inclusion of COX-2 status in prognostic models could refine risk stratification, allowing clinicians to prioritize follow-up and monitoring resources for patients at a higher likelihood of metastatic progression.

By focusing on COX-2, a broader view of downstream pathways involving various prostaglandins can be gained, potentially offering insights beyond PGE2 alone. Moreover, COX-2 inhibitors have shown potential therapeutic benefits for cancer treatment due to their anti-inflammatory properties, expanding the clinical relevance of targeting COX-2 [16]. The choice between COX-2 and PGE2 as a predictive marker should be determined by their respective correlations with CRC metastasis and practical factors such as cost and convenience of measurement [17]. If PGE2 is proven to be a more accurate indicator of metastasis, its ease of measurement might make it a compelling alternative, provided it aligns with clinical goals.

Alongside COX-2, emerging biomarkers like microRNAs, specific genetic mutations, and immune-related indicators are promising additions as prognostic indicators in CRC metastasis [51,52]. Integrating these markers with COX-2 could yield a more comprehensive view of disease progression. Advanced technologies such as omics-based approaches (genomics, transcriptomics, proteomics) offer intricate insights into CRC's molecular landscape, potentially refining prognostic models by identifying unique metastasis-associated signatures [[53], [54], [55]]. Additionally, liquid biopsies and artificial intelligence (AI) bring innovation – liquid biopsies enable non-invasive real-time tracking of tumor dynamics, while AI-driven analyses can unveil intricate patterns in omics data and clinical information, leading to more accurate prognostic predictions and tailored therapeutic strategies [[56], [57], [58]].

### 4.4 Clinical implications

If COX-2 is shown to be a strong prognostic factor, it may encourage the development and testing of targeted therapies specifically aimed at inhibiting COX-2 in colorectal cancer patients at high risk of metastasis. This could lead to the development of new treatment options. Secondly, by identifying COX-2 as a prognostic marker could potentially help increase survival rates by allowing for earlier intervention or more aggressive treatment for high-risk patients [4,10]. This, in turn, could positively impact the overall prognosis for colorectal cancer patients.

### 4.5 Potential future research directions

Conducting additional validation studies in diverse patient populations and settings can further confirm the prognostic value of COX-2 in colorectal cancer metastasis prediction [10]. This can help establish its robustness and generalizability. Future research can explore the potential benefits of combining COX-2 inhibitors with other treatment modalities, such as chemotherapy, immunotherapy, or targeted therapies. Combining treatments may yield synergistic effects in preventing metastasis [10]. Lastly, exploring how knowledge of COX-2 status affects patient-reported outcomes, quality of life, and treatment decision-making. Understanding the patient perspective is essential for translating research findings into meaningful clinical practice.

### 4.6 Limitations

There is a risk of publication bias due to the small number of papers included in this systematic review and meta-analysis but the study was novel and the immunohistochemistry staining process was extensive. There were few reports on COX-2 expression in CRC patients with metastasis and no studies provided cut-off points. However, significant effort was made to ensure that the analysis contained only reliable studies.

## 5 Conclusion

COX-2 expression is associated with lymph node invasion but not venous invasion in CRC. Further studies should determine the prognostic significance of COX-2 expression in determining metastasis status for CRC patients. Additional investigations in diverse populations are required to establish wider applicability, particularly concerning genetic variations among different groups [59].

## Source of funding

This research received no specific grant from any funding agency in the public, commercial, or not-for-profit sectors.

## Ethical statement

Not applicable.

## Data availability statement

The data presented in this study are openly available in Zenodo at: The Prognostic Value of COX-2 in Predicting Metastasis of Patients with Colorectal Cancer: A systematic review and meta analysis. https://doi.org/10.5281/zenodo.8388585 [60].

## CRediT authorship contribution statement

**Andriana Purnama:** Conceptualization, Data curation, Formal analysis, Investigation, Methodology, Resources, Supervision, Validation, Writing – original draft, Writing – review & editing. **Kiki Lukman:** Conceptualization, Data curation, Formal analysis, Investigation, Methodology, Resources, Supervision, Validation, Writing – original draft, Writing – review & editing. **Reno Rudiman:** Conceptualization, Data curation, Formal analysis, Investigation, Methodology, Resources, Supervision, Validation, Writing – original draft, Writing – review & editing. **Dwi Prasetyo:** Conceptualization, Data curation, Formal analysis, Investigation, Methodology, Resources, Supervision, Validation, Writing – original draft, Writing – review & editing. **Yoni Fuadah:** Conceptualization, Data curation, Formal analysis, Investigation, Methodology, Resources, Supervision, Validation, Writing – original draft, Writing – review & editing. **Prapanca Nugraha:** Conceptualization, Data curation, Formal analysis, Investigation, Methodology, Project administration, Software, Supervision, Validation, Writing – original draft, Writing – review & editing. **Valeska Siulinda Candrawinata:** Conceptualization, Data curation, Formal analysis, Investigation, Project administration, Resources, Software, Supervision, Validation, Writing – original draft, Writing – review & editing.

## Declaration of competing interest

The authors declare that they have no known competing financial interests or personal relationships that could have appeared to influence the work reported in this paper.
